# Exploring the causal association between genetically determined circulating metabolome and hemorrhagic stroke

**DOI:** 10.3389/fnut.2024.1376889

**Published:** 2024-05-15

**Authors:** Yaolou Wang, Yingjie Shen, Qi Li, Hangjia Xu, Aili Gao, Kuo Li, Yiwei Rong, Shang Gao, Hongsheng Liang, Xiangtong Zhang

**Affiliations:** ^1^Department of Neurosurgery, The First Affiliated Hospital of Harbin Medical University, Harbin, Heilongjiang, China; ^2^School of Life Science, Northeast Agricultural University, Harbin, Heilongjiang, China; ^3^NHC Key Laboratory of Cell Transplantation, Harbin, Heilongjiang, China

**Keywords:** causal association, circulating metabolome, hemorrhagic stroke, linkage disequilibrium score regression, Mendelian randomization

## Abstract

**Background:**

Hemorrhagic stroke (HS), a leading cause of death and disability worldwide, has not been clarified in terms of the underlying biomolecular mechanisms of its development. Circulating metabolites have been closely associated with HS in recent years. Therefore, we explored the causal association between circulating metabolomes and HS using Mendelian randomization (MR) analysis and identified the molecular mechanisms of effects.

**Methods:**

We assessed the causal relationship between circulating serum metabolites (CSMs) and HS using a bidirectional two-sample MR method supplemented with five ways: weighted median, MR Egger, simple mode, weighted mode, and MR-PRESSO. The Cochran Q-test, MR-Egger intercept test, and MR-PRESSO served for the sensitivity analyses. The Steiger test and reverse MR were used to estimate reverse causality. Metabolic pathway analyses were performed using MetaboAnalyst 5.0, and genetic effects were assessed by linkage disequilibrium score regression. Significant metabolites were further synthesized using meta-analysis, and we used multivariate MR to correct for common confounders.

**Results:**

We finally recognized four metabolites, biliverdin (OR 0.62, 95% CI 0.40–0.96, *P*_MVMR_ = 0.030), linoleate (18. 2n6) (OR 0.20, 95% CI 0.08–0.54, *P*_MVMR_ = 0.001),1-eicosadienoylglycerophosphocholine* (OR 2.21, 95% CI 1.02–4.76, *P*_MVMR_ = 0.044),7-alpha-hydroxy-3 -oxo-4-cholestenoate (7-Hoca) (OR 0.27, 95% CI 0.09–0.77, *P*_MVMR_ = 0.015) with significant causal relation to HS.

**Conclusion:**

We demonstrated significant causal associations between circulating serum metabolites and hemorrhagic stroke. Monitoring, diagnosis, and treatment of hemorrhagic stroke by serum metabolites might be a valuable approach.

## 1 Introduction

In recent studies, stroke has been considered the second leading cause of death (6.6 million persons) and disability [143 million Disability Life Years (DALYs) loss] worldwide. In the last three decades, the global incidence of stroke has increased by 70%, the prevalence of stroke has increased by 85%, the mortality rate has risen by 43%, and DALYs attributable to stroke have risen by 32%, amongst which hemorrhagic stroke (HS) is a major contributor (1, 2), there is an urgent need to explore the underlying molecular mechanisms of hemorrhagic stroke. HS is the deadliest form of stroke and includes subtypes of intracerebral hemorrhage (ICH) and subarachnoid hemorrhage (SAH) (3). Currently, neuroinflammation, oxidative stress, chronic damage to the vascular wall, cell death, and hemodynamic alterations have been discovered to be tightly related to the development of hemorrhagic stroke (4, 5). Thus, searching for the molecules that drive these microscopic mechanisms is particularly significant.

Circulating serum metabolites are a series of metabolic substrates and products closely associated with human health (6). Circulating serum metabolites (CSMs) drive various cellular functions, such as energy production and storage, signal transduction, and apoptosis (7). They can act in the development of a variety of diseases, such as chronic kidney disease, oncological diseases, and myocardial infarction (8–10). As for the nervous system, lipid metabolites with a function of triggering nerve repair were discovered (11), and a group of circulating serum metabolites consisting of serine, isoleucine, betaine, PC (5:0/5:0), and LysoPE (18:2) were judged to be excellent biomarkers for the diagnostic and progression process of acute ischemic stroke (AIS) (12). Also, a meta-analysis from seven prospective cohorts investigating the connections between serum or circulating metabolites and stroke risk found that the amino acid histidine, the glycolysis-related metabolite pyruvate, the acute-phase response marker glycoprotein acetyls, and several lipoprotein subfractions were linked to stroke risk. All stroke events, as well as ischemic and hemorrhagic events, were included in the analysis (13); likewise, after stroke onset can reduce human blood plasma metabolites such as branched-chain amino acids (valine, leucine, and isoleucine) that correlate with poor neurological outcomes (14). These studies reveal an inextricable link between CSMs and stroke.

Mendelian randomization (MR) is an application of instrumental variable analysis. It utilizes genetic variation to ascertain whether an observed association between risk factors and outcome corresponds to the causal effect. The principle refers to Mendel’s second law about the independent segregation of genetic alleles when DNA is passed from parents to offspring at gamete formation (15). Since MR analysis generates a random distribution of genetic variants through natural arbitrary classification, which is immutable, this method not only lowers the risk of confounding but also decreases the danger of reverse causality bias and is not influenced by disease state (16). Meanwhile, randomized controlled trials (RCTs) as the gold standard of causality have unavoidable problems such as substantial financial investment, weak patient compliance, and the ethics of randomized treatment allocation. Further, in situations where RCTs are unavailable, MR is extensively employed to infer a causal connection between exposure and outcome, with equally compelling results.

Based on the background mentioned above, we utilized MR analysis to detect the causal relationship between the circulating metabolome and hemorrhagic stroke to search for the metabolic pathways and mechanisms of effect behind the causality.

## 2 Materials and methods

### 2.1 Ethics statement and study design

This study did not require additional ethical approval since we used publicly available data, which had already received approval from the appropriate ethical and institutional review boards.

In this study, we evaluated the causal relationship between human blood metabolites and the risk of hemorrhagic stroke using a two-sample bidirectional MR design. A scientific MR study should comply with three hypotheses: (1) Instrumental variables (IVs) are strongly associated with the exposure of interest; (2) IVs must be independent of confounders; and (3) IVs are not related to the outcome, but only affect the outcome through exposure. And follow the Strengthening the Reporting of Observational Studies in Epidemiology Using Mendelian Randomization guidelines (STROBE-MR) (Supplementary Table S1) (17).

**Figure 1:**
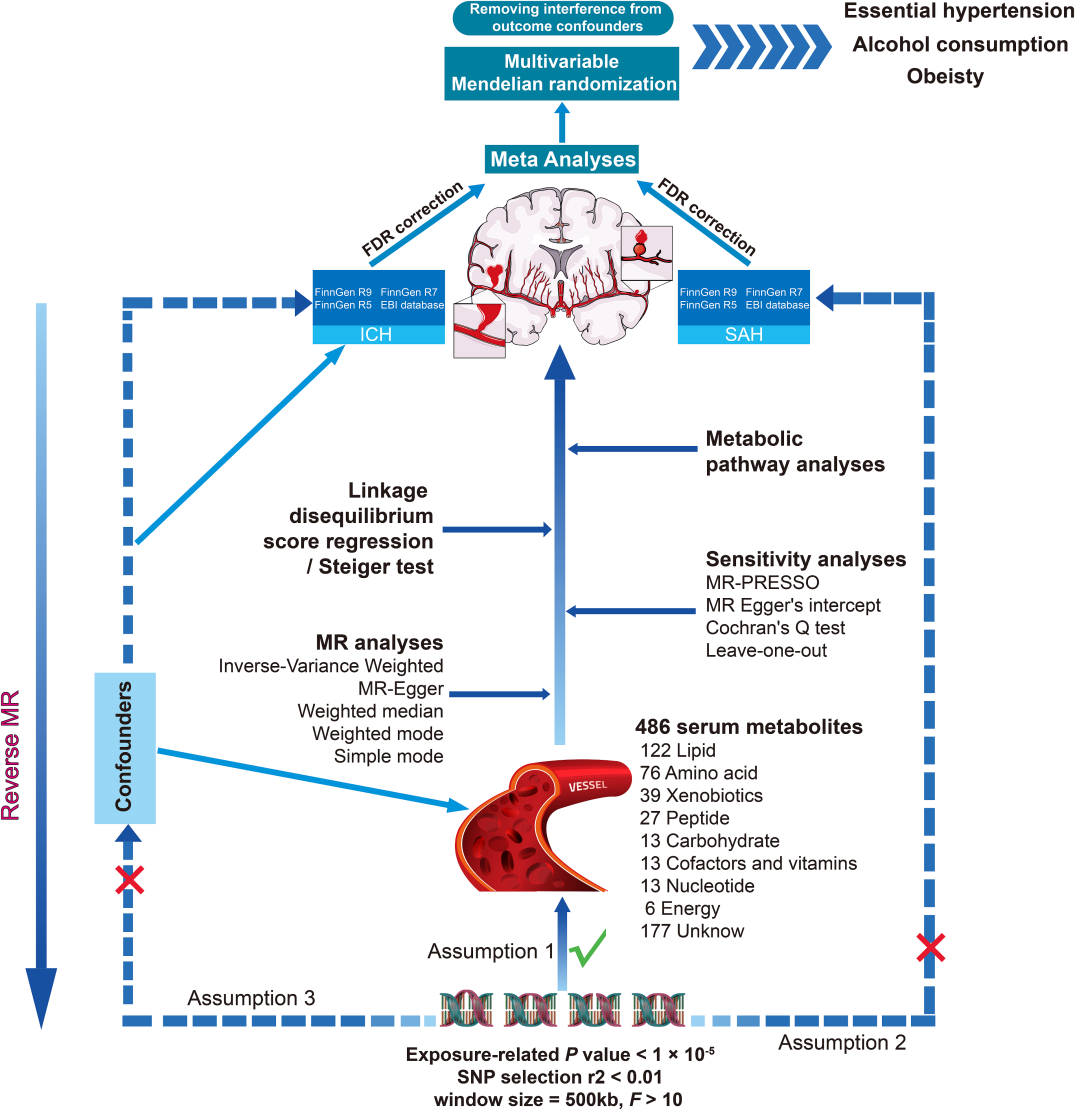
Overall study overview flowchart. SNP, single nucleotide polymorphism; MR, Mendelian randomization; ICH, intracerebral hemorrhage; SAH, subarachnoid hemorrhage; MR-PRESSO, MR pleiotropy residual sum and outlier.

Notably, we each used four different GWAS summary data to perform a comprehensive MR analysis with hemorrhagic stroke as the outcome. False discovery rate correction for *p*-value, meta-analysis, and multivariate Mendelian randomization were performed to increase the reliability and stability of the results, linkage disequilibrium score regression (LDSC) was used by us to assess the genetic correlation between the screened exposures and the outcome, and similarly, we also explored the potential mechanisms of circulating serum metabolites for hemorrhagic stroke by metabolite pathway analyses, as outlined in the present study in Figure 1.

### 2.2 Data source for circulating serum metabolites

We downloaded summary-type GWAS data for human serum metabolites from the Metabolomics GWAS Server.^1^ Notably, this is the most comprehensive report on the genetic loci of blood metabolites, which successfully screened out 486 metabolites with genetic influences on human serum metabolites by Shin et al. (18). Specifically, the study included 7,824 Europeans, including 1768 from the KORA F4 study in Germany and 6,056 from the United Kingdom Twin Study. This study analyzed 529 metabolites in plasma or serum from 7,824 adult individuals from two European population studies via liquid chromatography and gas chromatography separation-coupled tandem mass spectrometry. Four hundred and eighty-six serum metabolites were obtained after rigorous quality control. Among the 486 metabolites, 177 were defined as unknown due to poorly defined chemical properties. Another 309 metabolites were chemically authenticated and allocated to eight broad metabolic groups, including amino acid, carbohydrate, cofactors and vitamin, energy, lipid, nucleotide, peptide, and xenobiotic metabolism, as documented in the Kyoto Encyclopedia of Genes and Genomes (KEGG) database. We record additional information in Supplementary Table S2.

### 2.3 Data source for hemorrhagic stroke

Hemorrhagic stroke includes two subtypes, intracerebral hemorrhage (ICH) and subarachnoid hemorrhage (SAH). GWAS summary data for hemorrhagic stroke were obtained from the FinnGen and European Bioinformatics Institute (EBI) databases. For more detail, for ICH and SAH summary data, we chose the R9, R7, and R5 versions of the FinnGen database as well as the registry numbers downloaded from the GWAS Catalog for the data sets GCST90018870 and GCST90018923 datasets, the latter being the result of the meta-analysis of FinnGen R3 and UK Biobank by Saori Sakaue et al. (19). We show the specific features of the above datasets in Supplementary Table S2. More information can be acquired by accessing (https://www.finngen.fi/fi) and (https://www.ebi.ac.uk/gwas/).

### 2.4 Selection of instrumental variables

We selected instrumental variables to satisfy the three main assumptions mentioned above, and the first step was to screen SNPs for subsequent MR analyses by an association threshold of *p* < 1 × 10^−5^ to maximize the amount of genetic variance explained by genetic predictors (20). Second, SNPs were clustered in the European 1,000 Genomes Project Phase III reference panel using the R software with a linkage disequilibrium threshold *r*^2^ < 0.01 within 500 kilobases (kb), a condition that has been widely used in previous studies (21, 22). Finally, at the same time, the *F*-statistic was used as a reliable measure to assess the tool’s robustness. Ultimately, the F-statistic serves as a robust and reliable metric for evaluating the tool. We used the following formula to calculate:


$$F=\frac{R^{2}\left(n-1-k\right)}{\left(1-R^{2}\right)k}$$

Where *R*^2^ denotes explained variance, *n* denotes sample size, and *k* denotes the number of selected IVs.

### 2.5 Bidirectional MR analysis and sensitivity analysis

For univariable MR analysis, given that the random-effects inverse variance weighted (IVW) estimates were derived from a pooled analysis of Wald ratios across all genetic variants and the premise that IVW could provide the most accurate assessment of causal effects based on the assumption that there was no horizontal pleiotropy across all the included SNPs (23). We chose a *p* < 0.05 for the results of IVW as a preliminary assessment of a causal relationship between circulating serum metabolites (CSMs) and hemorrhagic stroke (HS). We also used five methods, Weighted median, MR Egger, Simple mode, Weighted mode, and MR-PRESSO, as complementary analyses to the IVW method, although the Weighted median method produces unbiased estimates even when as much as 50% of the data come from invalid instruments (24). The Weighted mode method is reliable when most individual instrument causal effect estimates come from valid instruments, even if some are considered invalid (25). Simple mode represents an unweighted empirical density function for causal estimation (26). In addition, the MR-Egger method is a valuable tool for estimating causal effects through the slope coefficients of Egger regressions, which helps to identify and address potential slight study bias (27). When the condition of IVW method *p* < 0.05, even though the *p* value of the five methods of supplementary analysis does not satisfy all <0.05, but the β value of all methods show the same direction effect, the result is recognized as positive circulating serum metabolites (28), which are included in the next stage of analysis. Meanwhile, the MR-PRESSO method results in a *p* < 0.05, which can further increase the accuracy and stability of positive results. As for the reverse MR analysis, we chose HS for exposure, significant and potentially causal CSM as the outcome, and applied the identical SNP selection conditions of the forward MR analysis by which to test the directionality of causality.

For the MR results of positive primary screening in the above steps, we corrected the *p* value of false discovery rate (FDR) according to the different types of HS and the different classifications of CSM to derive the corresponding *P*_FDR_. When the *p* < 0.05 and *P*_FDR_ < 0.1, we considered that there was a significant causative relationship between the exposure and outcome (29). Moreover, we thought of a potential causal relationship between exposure and outcome when *p* < 0.05 but *P*_FDR_ ≥ 0.1.

For sensitivity analyses of significant and potential causality, we employed three methods, Cochran’s Q test, MR-Egger intercept test, and MR-PRESSO, to identify horizontal pleiotropy and heterogeneity and to mitigate their effects by removing outliers. Cochran’s *Q*-test was used to detect heterogeneity, while the MR-Egger intercept test and MR-PRESSO (Global test) were used to detect horizontal pleiotropy of selected SNP, and the MR analysis was repeated after excluding these pleiotropic SNP. *p* > 0.05 indicates the absence of heterogeneity or pleiotropy (27, 30, 31). Additionally, to ensure unbiased causal estimation, we performed a leave-one-out analysis, which assesses whether the results are affected by the severity of a single SNP by discarding each SNP in turn and then performing MR analysis (32).

### 2.6 Linkage disequilibrium score and directionality tests

Although SNPs associated with HS were excluded during screening for IV, SNPs that can mediate the inheritance of HS still exist. And MR analyses may violate the causal effect in the presence of a genetic correlation between exposure and outcome (33). LDSC regression observes the genetic contribution of complex traits and characteristics by estimating the strength of association between SNPs and traits. Therefore, we utilized the LDSC to examine the genetic correlation between positive circulating serum metabolites and HS to ascertain that the genetic concordance of exposure and outcome did not confound the inter-causal effects. We also performed a Steiger test to eliminate bias due to reverse causality and validate causality’s directionality (34), providing a more informative interpretation of the reverse MR results.

### 2.7 Metabolic pathway analysis

We explored the possible pathways and mechanisms of action behind circulating serum metabolites with significant and potentially causal effects on hemorrhagic stroke using MetaboAnalyst 5.0^2^ (35).

### 2.8 Meta-analysis and multivariate MR analysis

Aiming to make the final screening of circulating serum metabolites causally associated with hemorrhagic stroke comprehensive, precise, and constant, ICH and SAH in hemorrhagic stroke, we used four different GWAS data each. Then, a meta-analysis of the results of the IVW model was carried out using the R software package, followed by the screening of the corresponding *P*_Meta_, with *P*_Meta_ > 0.05 removed. Meanwhile, we applied *I*^2^ to perform the heterogeneity test of meta-results (36).

To adequately adjust for confounders, circulating serum metabolites after meta-analysis selection were further included as confounders (hypertension, obesity, alcohol consumption) in our MR analysis for multivariate MR analysis (MVMR). IVW, weighted median, and MR-Egger regression were also analyzed in the MVMR analysis. Besides, Egger-intercept and Cochran’s *Q* tests were evaluated to assess the multiplicity and heterogeneity of the results (37).

### 2.9 Analysis software and packages

All the statistical analyses and data visualizations were performed using R software version 4.3.1. Bidirectional and multivariate MR analyses were implemented with the packages “TwoSampleMR” (26), “MR-PRESSO” (31), and “MendelianRandomization” (38); meta-analysis was done using the “meta” (39) package.

## 3 Results

### 3.1 Instrumental variables selection in causal analyses

Following rigorous instrumental variable selection rules and procedures, we performed MR analyses on 309 known circulating serum metabolites, acquiring IVs ranging from 3 to 485 SNPs. All selected SNPs had *F* values exceeding 10, showing instrumental solid efficacy.

### 3.2 Causal assessment of circulating metabolome leading to hemorrhagic stroke

**Figure 2:**
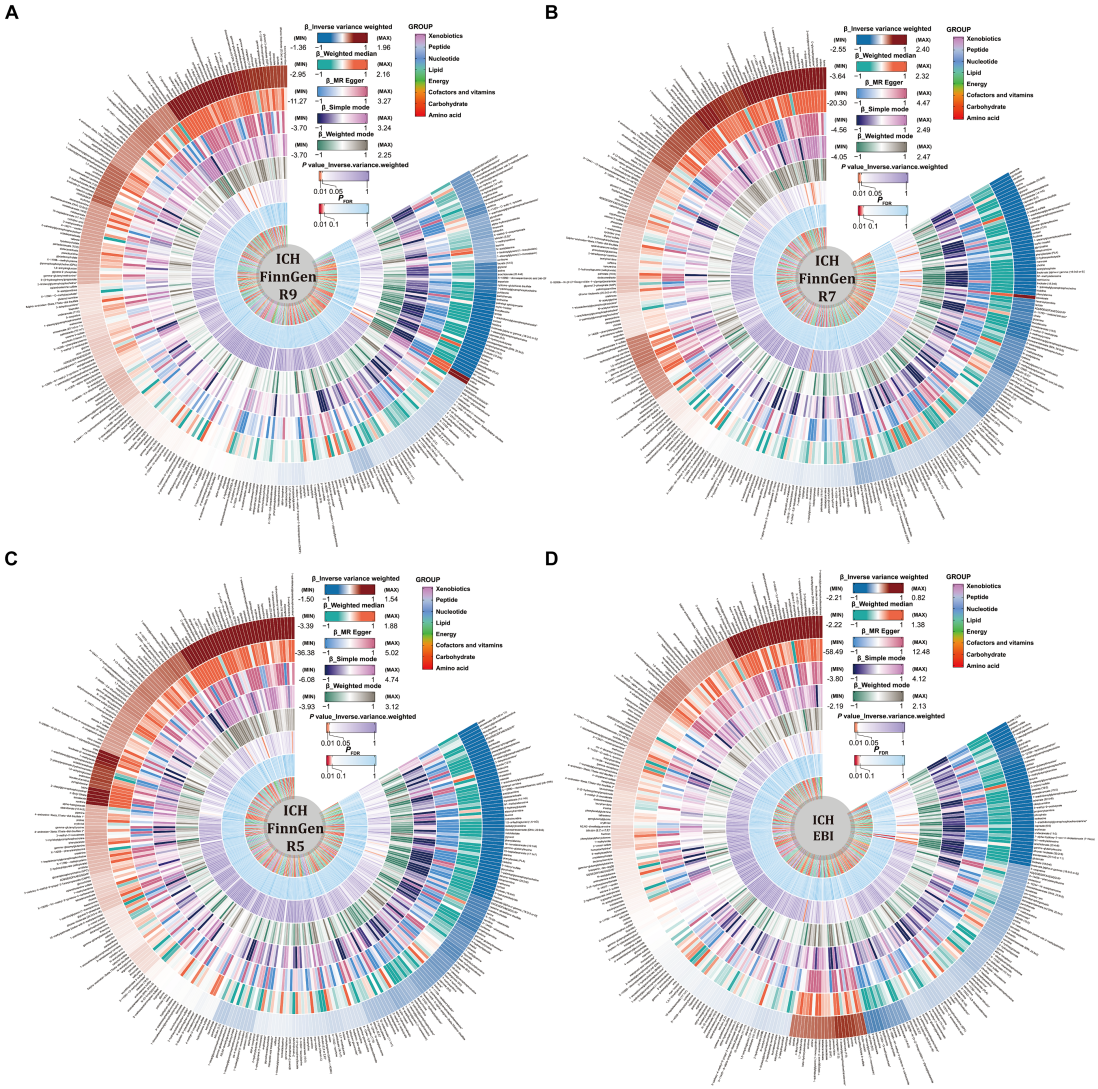
Causal relationships between circulating serum metabolites and intracerebral hemorrhage based on different data versions; respectively **(A)** FinnGen R9 **(B)** FinnGen R7 **(C)** FinnGen R5 **(D)** EBI database. From outside to inside, the β values of the five methods of inverse variance weighted (IVW), weighted median, MR egger, simple mode, weighted mode, the *p* values of IVW model, the *P*_FDR_, and the metabolite species grouping are shown sequentially.

**Figure 3:**
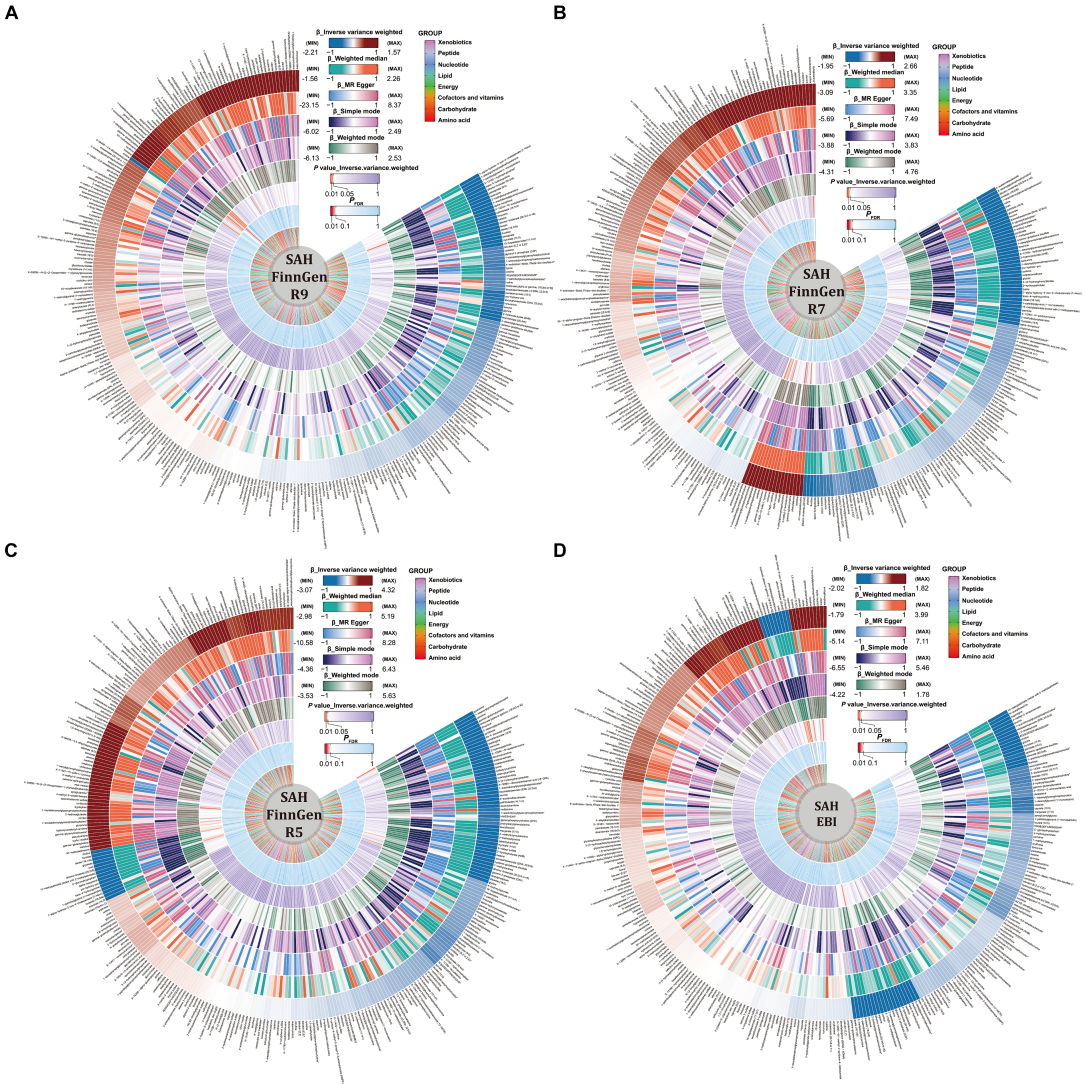
Causal relationships between circulating serum metabolites and subarachnoid hemorrhage based on different data versions; respectively **(A)** FinnGen R9 **(B)** FinnGen R7 **(C)** FinnGen R5 **(D)** EBI database. From outside to inside, the β values of the five methods of inverse variance weighted (IVW), weighted median, MR egger, simple mode, weighted mode, the *p* values of IVW model, the *P*_FDR_, and the metabolite species grouping are shown sequentially.

Detailed results of the IVs and each method selected for positive MR analysis of 309 CSMs of known structure and function with HS are shown in their entirety in Supplementary Tables S3, S4, and the visualization of the results is available in Figures 2, 3. We judged that CSMs with IVW model *p* < 0.05 were positive for the presence of a causal effect, provided that the β values of the five analytical methods were in the same direction, and *P*_FDR_ < 0.1 were considered to have a significant causal effect.

As for the ICH results, following the above selection criteria, we identified a total of 39 positive CSMs in seven significant categories for the four ICH GWAS data, classified according to circulating serum metabolites, of which 18 were in Lipid, 9 in Amino acid, 3 in Peptide, 3 in Cofactors and vitamins, 3 in Xenobiotics, 1 in Carbohydrate, and 2 in Nucleotide. Corresponding β, OR, and *p* values can be searched in Supplementary Table S4 and Figure 2.

For the SAH results, with the same screening standard, we recognized 34 positive CSMs in six major categories for the four SAH GWAS data, according to the circulating serum metabolite classification, of which 18 belong to Lipid, 8 to Amino acid, 4 to Peptide, 2 to Carbohydrate, 1 to Cofactors and vitamins and 1 to Nucleotide. Matching β, OR, and *p* values are shown in Supplementary Table S2 and Figure 3.

**Figure 4:**
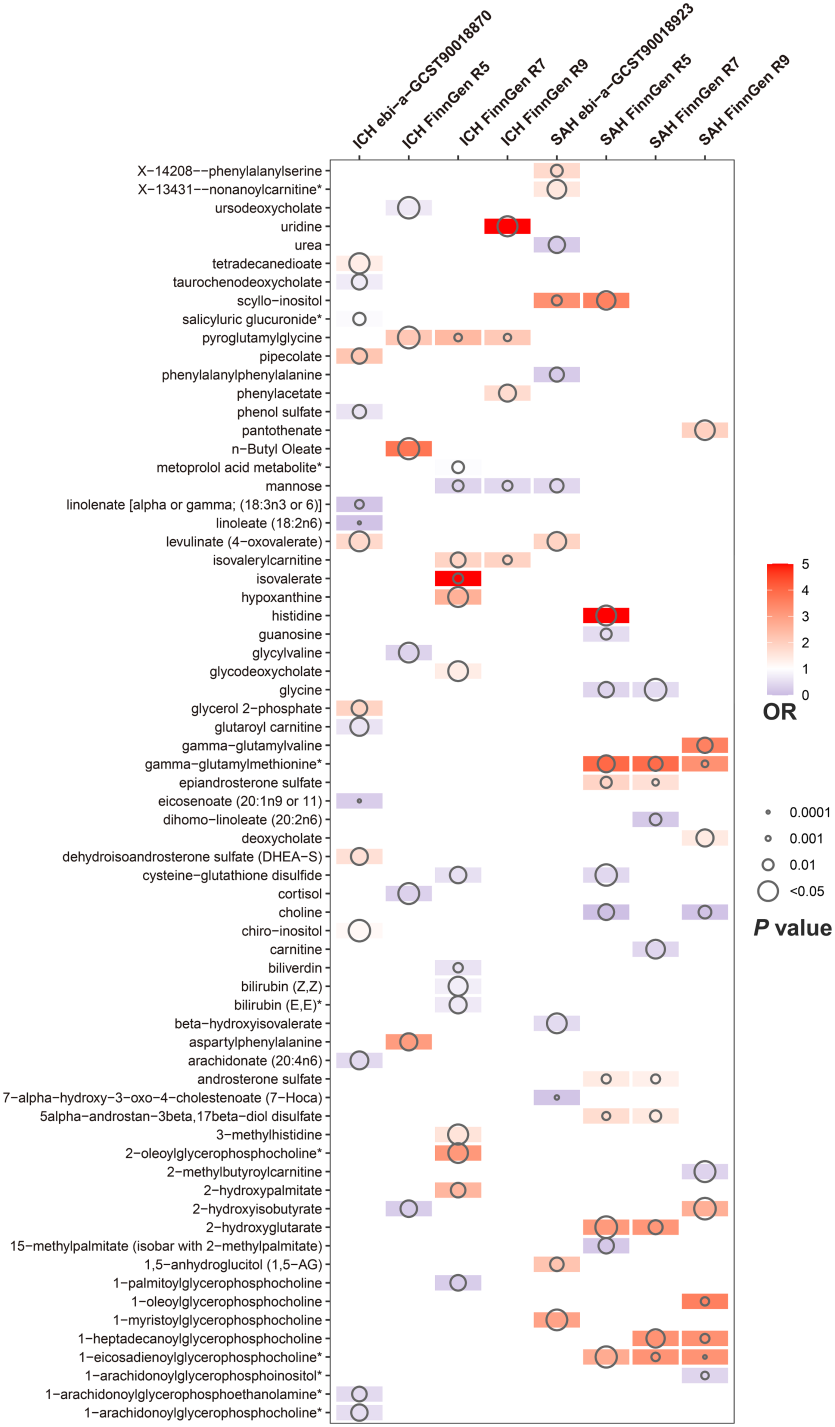
Circulating serum metabolites with causal effects on outcome in different versions of hemorrhagic stroke. Demonstrate the magnitude of ORs and *p*-values based on the IVW model. OR, odds ratio; IVW, inverse-variance weighted.

**Figure 5:**
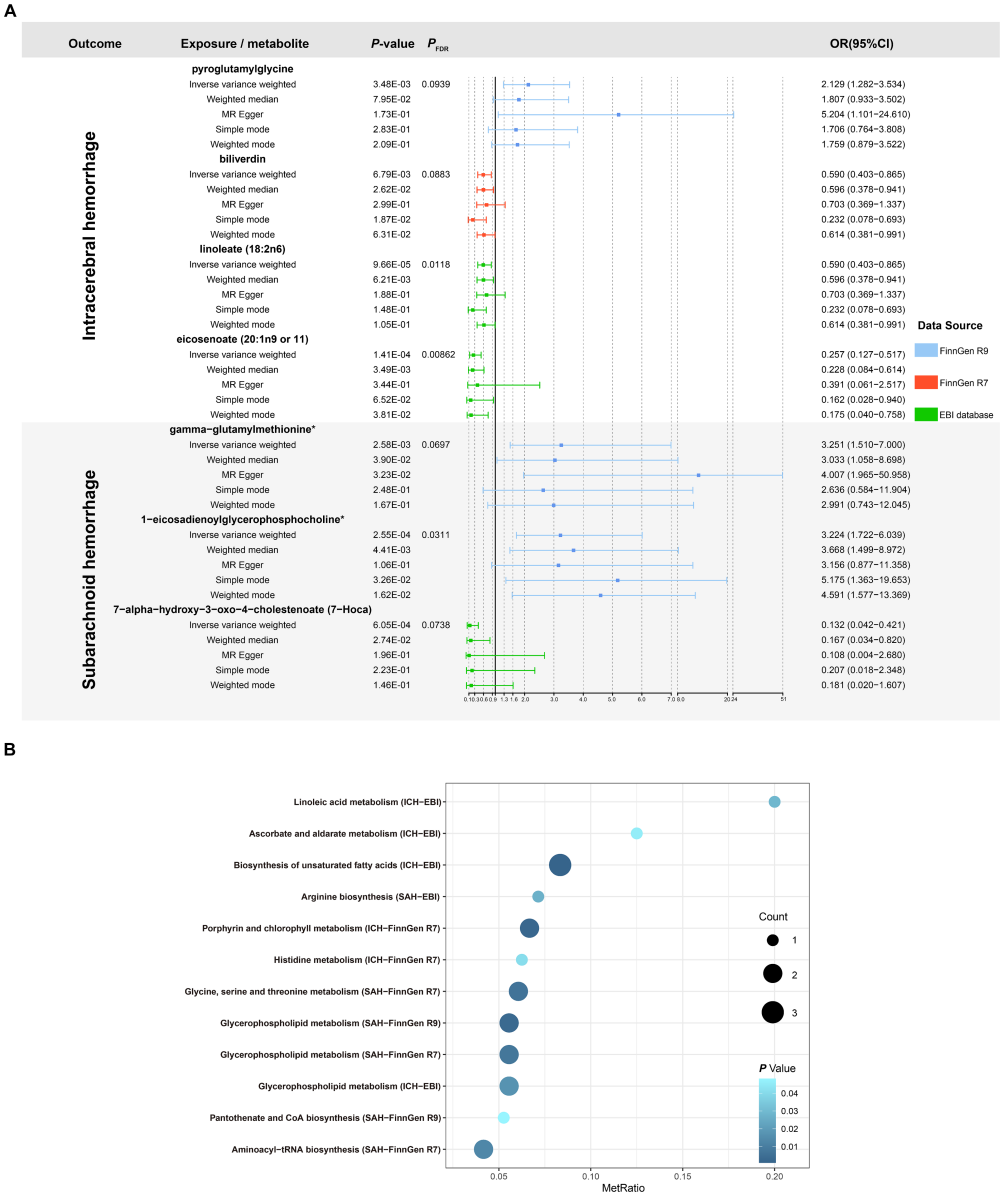
**(A)** Forest plot of five MR analysis methods for circulating serum metabolites significantly and causally associated with hemorrhagic stroke. **(B)** Significant metabolic pathways enriched based on different versions of hemorrhagic stroke outcome. MetRatio = Metabolites enriched/Total number of metabolites in the metabolic pathway.

Of the above 73 positive circulating serum metabolites after rectification (Figure 4), 7 of these (*P*_FDR_ < 0.1) were determined to be significant causative CSMs, ICH was the outcome for 4, namely pyroglutamylglycine in Peptide (OR 2.13, 95% CI 1.28–3.53, *p* = 0.00348, *P*_FDR_ = 0.0939), biliverdin in Cofactors and vitamins (OR 0.59, 95% CI 0.40–0.86, *p* = 0.00679, *P*_FDR_ = 0.0883), linoleate (18:2n6) in Lipid (OR 0.11, 95% CI 0.04–0.33, *p* = 0.0000966, *P*_FDR_ = 0.0118) and eicosenoate (20:1n9 or 11) (OR 0.26, 95% CI 0.13–0.52, *p* = 0.000141, *P*_FDR_ = 0.00862) in Lipid, and 3 for SAH as an ending, for gamma-glutamylmethionine* in Peptide(OR 3.25, 95% CI 1.51–7.00, *p* = 0.00258, *P*_FDR_ = 0.0697), 1-eicosadienoylglycerophosphocholine* in Lipid (OR 3.22, 95% CI 1.72–6.04, *p* = 0.000255, *P*_FDR_ = 0.0311) and 7-alpha-hydroxy-3-oxo-4-cholestenoate (7-Hoca) in Lipid (OR 0.13, 95% CI 0.04–0.42, *p* = 0.000605, *P*_FDR_ = 0.0738). The above results are presented in Table 1 and Figures 2–4, 5A.

**Table 1:** Results of MR estimation of circulating serum metabolites significantly and causally associated with hemorrhagic stroke.

Hemorrhagic stroke/outcome	Classification	Serum metabolites/exposure	Resulting source dataset	nSNP	Method of MR	β	SE	*p*-value	OR (95%CI)	*P*_FDR_
Intracerebral hemorrhage	Peptide	Pyroglutamylglycine	FinnGen R9	4	Inverse variance weighted	0.756	0.259	3.48E-03	2.129 (1.282–3.534)	9.39E-02
					Weighted median	0.592	0.337	7.95E-02	1.807 (0.933–3.502)	
					MR Egger	1.649	0.793	1.73E-01	5.204 (1.101–24.610)	
					Simple mode	0.534	0.410	2.83E-01	1.706 (0.764–3.808)	
					Weighted mode	0.565	0.354	2.09E-01	1.759 (0.879–3.522)	
	Cofactors and vitamins	Biliverdin	FinnGen R7	17	Inverse variance weighted	−0.528	0.195	6.79E-03	0.590 (0.403–0.865)	8.83E-02
					Weighted median	−0.517	0.232	2.62E-02	0.596 (0.378–0.941)	
					MR Egger	−0.353	0.328	2.99E-01	0.703 (0.369–1.337)	
					Simple mode	−1.461	0.558	1.87E-02	0.232 (0.078–0.693)	
					Weighted mode	−0.487	0.244	6.31E-02	0.614 (0.381–0.991)	
	Lipid	Linoleate (18:2n6)	ebi-a-GCST90018870	17	Inverse variance weighted	−2.206	0.566	9.66E-05	0.590 (0.403–0.865)	1.18E-02
					Weighted median	−2.220	0.811	6.21E-03	0.596 (0.378–0.941)	
					MR Egger	−2.988	2.166	1.88E-01	0.703 (0.369–1.337)	
					Simple mode	−2.108	1.388	1.48E-01	0.232 (0.078–0.693)	
					Weighted mode	−2.191	1.276	1.05E-01	0.614 (0.381–0.991)	
		Eicosenoate (20:1n9 or 11)		13	Inverse variance weighted	−1.360	0.357	1.41E-04	0.257 (0.127–0.517)	8.62E-03
					Weighted median	−1.481	0.507	3.49E-03	0.228 (0.084–0.614)	
					MR Egger	−0.938	0.950	3.44E-01	0.391 (0.061–2.517)	
					Simple mode	−1.822	0.898	6.52E-02	0.162 (0.028–0.940)	
					Weighted mode	−1.746	0.749	3.81E-02	0.175 (0.040–0.758)	
Subarachnoid hemorrhage	Peptide	Gamma-glutamylmethionine*	FinnGen R9	8	Inverse variance weighted	1.179	0.391	2.58E-03	3.251 (1.510–7.000)	6.97E-02
					Weighted median	1.110	0.538	3.90E-02	3.033 (1.058–8.698)	
					MR Egger	2.303	0.830	3.23E-02	10.007 (1.965–50.958)	
					Simple mode	0.969	0.769	2.48E-01	2.636 (0.584–11.904)	
					Weighted mode	1.096	0.711	1.67E-01	2.991 (0.743–12.045)	
	Lipid	1-Eicosadienoylglycerophosphocholine*		13	Inverse variance weighted	1.171	0.320	2.55E-04	3.224 (1.722–6.039)	3.11E-02
					Weighted median	1.300	0.456	4.41E-03	3.668 (1.499–8.972)	
					MR Egger	1.149	0.653	1.06E-01	3.156 (0.877–11.358)	
					Simple mode	1.644	0.681	3.26E-02	5.175 (1.363–19.653)	
					Weighted mode	1.524	0.545	1.62E-02	4.591 (1.577–13.369)	
		7-Alpha-hydroxy-3-oxo-4-cholestenoate (7-Hoca)	ebi-a-GCST90018923	16	Inverse variance weighted	−2.021	0.589	6.05E-04	0.132 (0.042–0.421)	7.38E-02
					Weighted median	−1.789	0.811	2.74E-02	0.167 (0.034–0.820)	
					MR Egger	−2.226	1.639	1.96E-01	0.108 (0.004–2.680)	
					Simple mode	−1.575	1.239	2.23E-01	0.207 (0.018–2.348)	
					Weighted mode	−1.710	1.114	1.46E-01	0.181 (0.020–1.607)	

SE, standard error; OR, odds ratio; SNP, single nucleotide polymorphism; FDR, false discovery rate.

### 3.3 Sensitivity tests in positive causal analysis

We did multiplicity and heterogeneity tests and leave-one-out analyses in the sensitivity analyses. MR-Egger regression intercepts were not significantly different from zero (all *P*_egger-intercept_ > 0.05) except for gamma-glutamylmethionine (*P*_egger-intercept_ = 0.046) for Peptide in FinnGen R7 SAH, in which the resulting causality was not plausible since there was horizontal multiplicity in this serum substance. The Cochran’s Q test for heterogeneity *p-*values were all more than 0.05; after removing all outliers of X-14208-phenylalanylserine from Peptide in EBI SAH MR-PRESSO Global test P- values were more than 0.05, without significant heterogeneity and pleiotropy (Supplementary Tables S5, S6). Moreover, leave-one-out analysis demonstrated that no particular SNPs driving the correlation between CSMs and HS existed (Supplementary Table S7).

### 3.4 Genetic association assessment and directionality test for positive causality

Genetic correlation through linkage disequilibrium (LDSC), when assessing causality using circulating serum metabolites as exposures, indicated that 3-methylhistidine (*R*g = 1.199, SE_Rg_ = 0.543, *P*_Rg_ = 0.027) as the outcome of FinnGen R7 ICH, ursodeoxycholate (*R*g = 1.026, SE_Rg_ = 0.475, *P*_Rg_ = 0.031) as the outcome of FinnGen R5 ICH, gamma-glutamylvaline (*R*g = 0.580, SE_Rg_ = 0.285, *P*_Rg_ = 0.042) as the outcome of FinnGen R9 SAH, guanosine (*R*g = 1.441, SE_Rg_ = 0.725, *P*_Rg_ = 0.047) as the outcome of FinnGen R5 ICH, These four metabolites were genetically correlated with the outcome meaning that the results of the MR analysis mixed and coexisted with the genetic component, so the initially gained causality between them was not sufficiently robust, as well as some of the metabolites for which the genetic correlation was calculated to be negative, which we denoted as NA (Supplementary Table S8). Also, the directionality check Steiger test results implied that none of the reverse causality effects influenced the forward causality (Supplementary Table S5).

### 3.5 Alterations in the circulating metabolome after hemorrhagic stroke

Reverse MR analyses involving circulating serum metabolites as outcome to estimate causality included FinnGen R7 ICH as exposure to mannose (β 0.026, 95% CI 0.008–0.044, *p* = 0.004, *P*_FDR_ = 0.065), EBI ICH as exposure to taurochenodeoxycholate (β 0.063, 95% CI 0.016–0.109, *p* = 0.008, *P*_FDR_ = 0.067) and tetradecanedioate with EBI ICH as the exposure (β 0.047, 95% CI 0.013–0.080, *p* = 0.007, *P*_FDR_ = 0.110) were considered to have a causal effect, whereby the levels of the mentioned three metabolites increased after the occurrence of ICH (Supplementary Tables S9, S10). Also, we did not find heterogeneity and pleiotropy, nor were SNPs significantly correlated with the results in leave-one-out analysis (Supplementary Tables S11, S12).

### 3.6 Effect pathway analysis of the circulating metabolome

By performing metabolic pathway analyses on GWAS data from 8 ICH and SAH as outcomes probing for causality, we characterized altogether 10 possible pathways of effect (*p* < 0.05), “Biosynthesis of unsaturated fatty acids” (*p* = 0.00088), “Linoleic acid metabolism” (*p* = 0.02873), “Ascorbate and aldarate metabolism” (*p* = 0.04562), “Porphyrin and chlorophyll metabolism” (*p* = 0.00212), and “Histidine metabolism” (*p* = 0.04069) might be the pathway mechanisms by which circulating serum metabolites contribute to the progression of ICH, while “Arginine biosynthesis” (*p* = 0.02687), “Glycine, serine and threonine metabolism” (*p* = 0.00625), “Aminoacyl-tRNA biosynthesis” (*p* = 0.01301), “Pantothenate and CoA biosynthesis “(*p* = 0.04818) may be related to the SAH disease process, “Glycerophospholipid metabolism” (*p* = 0.01705 for EBI ICH, *p* = 0.00742 for FinnGen R7 SAH, *p* = 0.00306 for FinnGen R9 SAH) is a probable metabolite action pathway common to the development of ICH and SAH (Supplementary Table S13 and Figure 5B).

### 3.7 Meta-analyses based on multiple versions of hemorrhagic stroke

**Figure 6:**
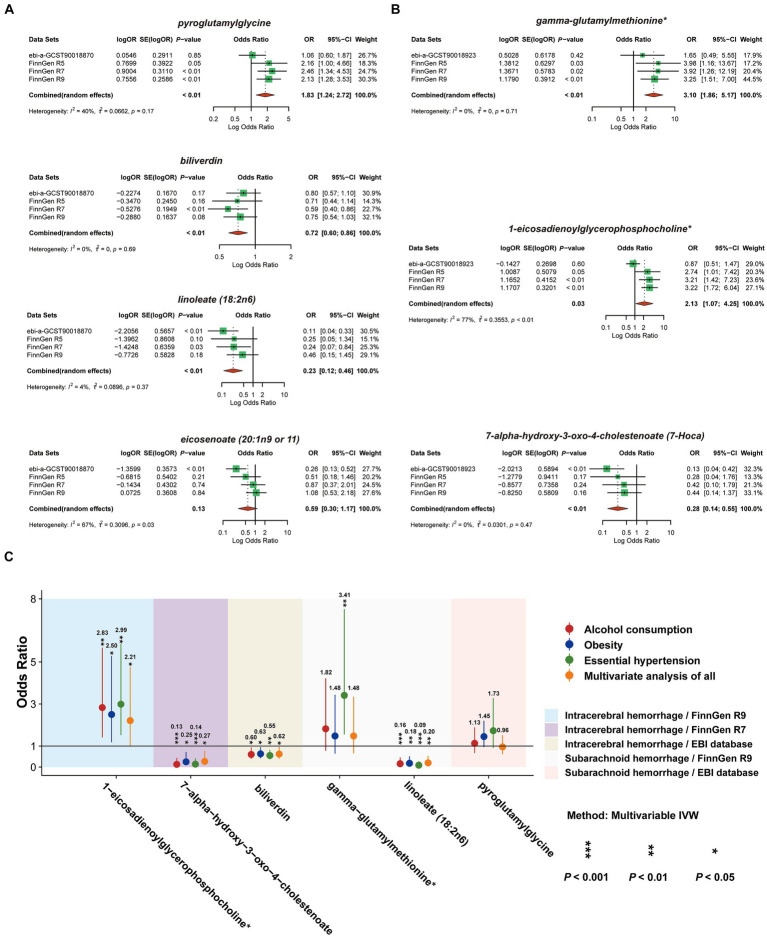
Meta-analysis of circulating serum metabolites with significant causality based on four versions of the outcome IVW model. Of these, **(A)** with ICH as the outcome **(B)** with SAH as the outcome. **(C)** De-confounded multivariate MR analysis incorporating alcohol consumption, obesity, and essential hypertension.

Aiming to further refine the exploration and evaluation of causal effects, we conducted a meta-analysis of the IVW model for each of the seven CSMs with significant causal associations with HS based on the results of the MR analyses of the four outcome data we selected, revealing that the results of biliverdin (OR 0.72, 95% CI 0.60–0.86, *P*_random effects_ < 0.01, *I*^2^ = 0%), pyroglutamylglycine (OR 1.83, 95% CI 1.24–2.72, *P*_random effects_ < 0.01, *I*^2^ = 40%), linoleate (18:2n6) (OR 0.23, 95% CI 0.12–0.46, *P*_random effects_ < 0.01, *I*^2^ = 4%) with a constant causal relationship between ICH, 1-eicosadienoylglycerophosphocholine* (OR 2.13, 95% CI 1.07–4.25, *P*_random effects_ = 0.03, *I*^2^ = 77%) (*I*^2^ suggests that there is heterogeneity in this result, which needs to be interpreted with caution), gamma- glutamylmethionine* (OR 3.10, 95% CI 1.86–5.17, *P*_random effects_ < 0.01, *I*^2^ = 0%), 7-alpha-hydroxy-3-oxo-4-cholestenoate (7-Hoca) (OR 0.28, 95% CI 0.14–0.55, *P*_random effects_ < 0.01, *I*^2^ = 0%) stablely influenced the progression of SAH, while eicosenoate (20:1n9 or 11) (OR 0.59, 95% CI 0.30–1.17, *P*_random effects_ = 0.13, *I*^2^ = 67%) with ICH causality was not plausible, thus it will be excluded from the subsequent analysis (Figures 6A,B).

### 3.8 Removing confounding effects with multivariate MR analysis

**Figure 7:**
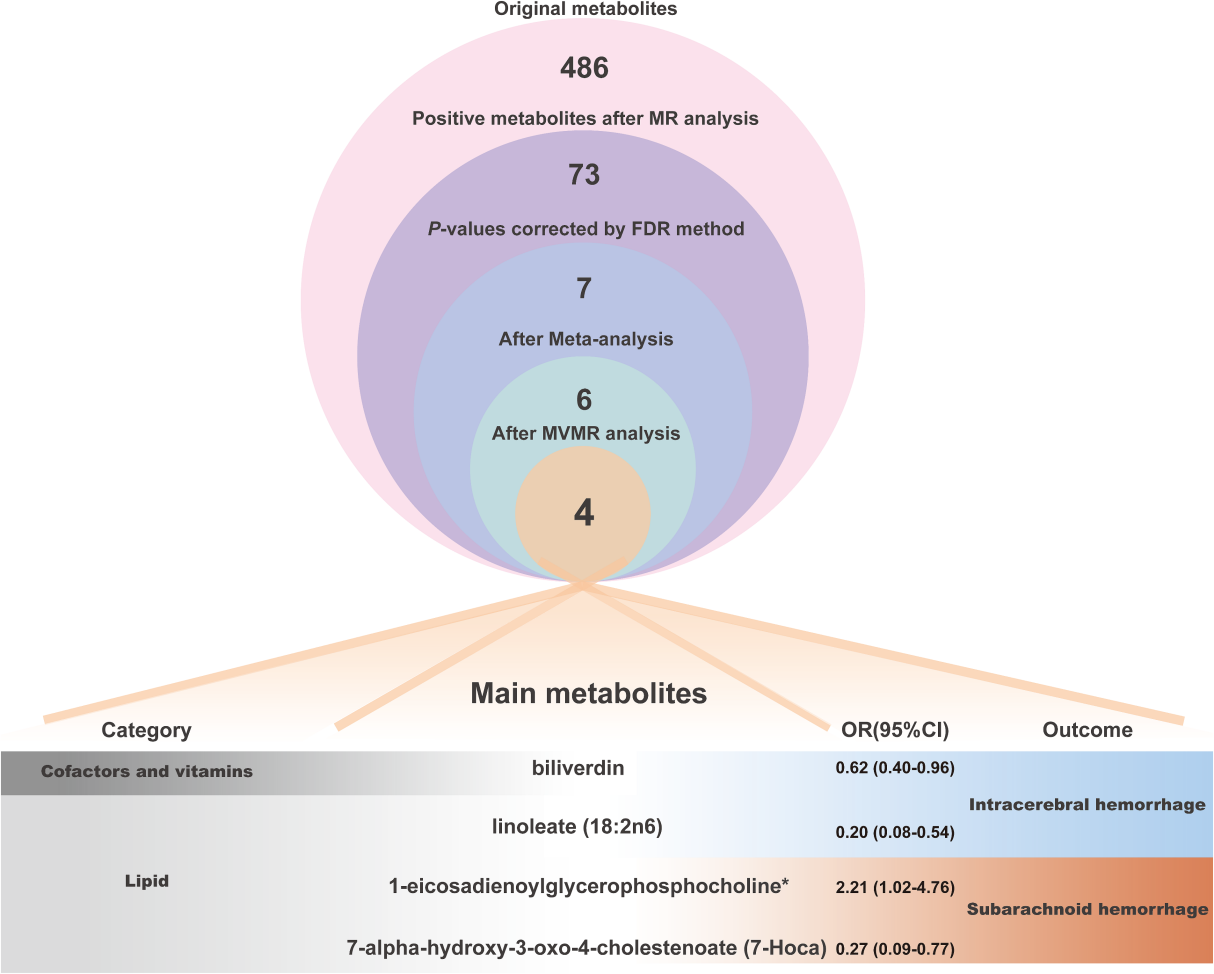
The analytical procedure and detailed information (category, OR values and outcome of action) of the 4 main metabolites were eventually obtained.

To ensure that the eventually obtained CSMs directly influenced HS rather than through confounders, we incorporated alcohol consumption, hypertension, and obesity as confounders, using the respective types of HS data as the outcome and performing multivariate MR analyses considering them separately and collectively. The results of the separate analyses are presented in Supplementary Table S14, and the results of the collective studies are shown in Table 2 and Figure 6C. In summary, four CSMs were identified that directly contributed to HS, of which biliverdin belonging to Cofactors and vitamins and linoleate (18:2n6) belonging to lipid were potential protective factors for ICH, while 1-eicosadienoylglycerophosphocholine* belonging to lipid and 7- alpha-hydroxy-3-oxo-4-cholestenoate (7-Hoca) belonging to lipid, the former being a possible risk factor for SAH and the latter a potential protective factor for SAH (Figure 7). We similarly conducted the Cochran’s Q test for MVMR analysis (Multivariable IVW and Multivariable MR-Egger) and MR-Egger’s intercept without potential heterogeneity and multiplicity (Supplementary Table S15).

**Table 2:** Results of multivariable MR estimation of circulating serum metabolites and hemorrhagic stroke.

Type of outcome	Classification	Type/metabolism	nSNP	Methods of multivariable MR	β	SE	*p*-value	OR (95%CI)
Intracerebral hemorrhage/FinnGen R9	Peptide	Pyroglutamylglycine	101	Multivariable IVW	−0.037	0.232	8.73E-01	0.96 (0.61–1.52)
				Multivariable median	0.164	0.303	5.89E-01	1.18 (0.65–2.13)
				Multivariable egger	0.002	0.234	9.93E-01	1.00 (0.63–1.59)
Intracerebral hemorrhage/FinnGen R7	Cofactors and vitamins	Biliverdin	114	Multivariable IVW	−0.476	0.219	3.01E-02	0.62 (0.40–0.96)
				Multivariable median	−0.510	0.255	4.55E-02	0.60 (0.36–0.99)
				Multivariable egger	−0.448	0.220	4.19E-02	0.64 (0.42–0.98)
Intracerebral hemorrhage/EBI database	Lipid	Linoleate (18:2n6)	105	Multivariable IVW	−1.599	0.503	1.48E-03	0.20 (0.08–0.54)
				Multivariable median	−1.641	0.699	1.88E-02	0.19 (0.05–0.76)
				Multivariable egger	−1.540	0.507	2.38E-03	0.21 (0.08–0.58)
Subarachnoid hemorrhage/FinnGen R9	Peptide	Gamma-glutamylmethionine*	105	Multivariable IVW	0.391	0.417	3.47E-01	1.48 (0.65–3.35)
				Multivariable median	0.066	0.513	8.98E-01	1.07 (0.39–2.92)
				Multivariable egger	0.413	0.416	3.21E-01	1.51 (0.67–3.42)
Subarachnoid hemorrhage/FinnGen R9	Lipid	1-Eicosadienoylglycerophosphocholine*	108	Multivariable IVW	0.792	0.392	4.35E-02	2.21 (1.02–4.76)
				Multivariable median	1.389	0.506	6.07E-03	4.01 (1.49–10.81)
				Multivariable egger	0.840	0.391	3.17E-02	2.32 (1.08–4.99)
Subarachnoid hemorrhage/EBI database		7-Alpha-hydroxy-3-oxo-4-cholestenoate (7-Hoca)	119	Multivariable IVW	−1.322	0.543	1.48E-02	0.27 (0.09–0.77)
				Multivariable median	−1.461	0.740	4.83E-02	0.23 (0.05–0.99)
				Multivariable egger	−1.322	0.543	1.48E-02	0.27 (0.09–0.77)

SE, standard error; OR, odds ratio; SNP, single nucleotide polymorphism.

## 4 Discussion

For this study, we selected to explore the causal associations between 309 known CSMs and HS by using eight different large GWAS datasets on hemorrhagic stroke. We performed several bidirectional two-sample MR analyses, combined with robustness checks of the results, after correction for *p*-values, Pyroglutamylglycine (Peptide), biliverdin (Cofactors and vitamins), linoleate (18:2n6) (Lipid), eicosenoate (20:1n9 or 11) (Lipid), gamma- glutamylmethionine*(Peptide), 1-eicosadienoylglycerophosphocholine* (Lipid), 7-alpha-hydroxy-3-oxo-4-cholestenoate (7-Hoca) (Lipid), these 7 CSMs were recognized as having a significant causal link with HS. Subsequently, we made use of meta-analysis to thoroughly investigate the causal relationships of these CSMs with distinct versions of hemorrhagic stroke. We found that the relevance of eicosenoate (20:1n9 or 11) (Lipid) to the outcome became insignificant. Moreover, we conducted multivariate MR analysis to remove potential confounders. Ultimately, we identified four CSMs that had significant causality with HS. Specifically, biliverdin (Cofactors and vitamins) and linoleate (18:2n6) (lipid) can serve as protective factors for ICH, and increased levels of 1-eicosadienoylglycerophosphocholine* (lipid) raise the risk of SAH. In contrast, high levels of 7-alpha-hydroxy-3-oxo-4-cholestenoate (7-Hoca) (lipid) lower the risk of SAH. Besides, we also appraised the genetic correlation through LDSC from CSMs and HS to clarify the coexistence of inherited effects, strengthening the reliability of our findings. Finally, metabolic pathway analysis was applied to find ten potential action pathways binding the two, including a shared path (glycerophospholipid metabolism) that CSMs act on both ICH and SAH. In recent similar studies, Wang et al. (40) focused on the effect of selected serum metabolites produced by gut flora on stroke, and our attention is centered on the role of the more comprehensive circulating metabolome on the mechanisms of hemorrhagic stroke. Zhang et al. conducted a broad and general exploration of the causal relationship between serum metabolites and various subtypes of stroke, including hemorrhagic and ischemic stroke (41). While existing studies lack in-depth studies on each of these subtypes, our research focusing on hemorrhagic stroke fills this gap. We used multiple versions rather than a single version of the ending data to comprehensively analyze the causality, then combined the results of the individual versions utilizing meta-analysis, followed by the MVMR method to remove the interference of common confounders associated with HS, and the interlocking analyses led us to obtain a wealthy and robust causal relationship between CSMs and HS. We believe that the circulating serum metabolites discovered in this comprehensive study can offer assistance in the prevention, treatment, and deeper mechanistic investigation of hemorrhagic stroke.

Recently, a large cohort study that explored the association of L-alpha glycerylphosphorylcholine (α-GPC) with the risk of subsequent stroke at 10 years noted a higher hazard of hemorrhagic stroke in α-GPC users compared to non-users, after adjusting for traditional cerebrovascular risk factors (42). This indicates that exogenous metabolites can influence the occurrence of HS in the form of altered intake in a dose–response manner. In a meta-analysis of 26 prospective cohort studies and 12 randomized controlled trials, fish and long chain omega 3 fatty acids intake were found to be moderately negatively related to cerebrovascular risk (43). Fish can supply a wealth of diverse nutrients which, upon entering the body, make their transformation into a variety of metabolites entering the circulatory system and thereby intervening in the occurrence of cerebrovascular events, suggesting that HS is also capable of being interfered with by variations in the categories and concentrations of circulating metabolites due to food intake. As for endogenous circulating metabolites, current findings demonstrate that greater circulating concentrations of vitamin D correlate with a lower incidence of cerebrovascular disease, whereas higher levels of circulating calcium relate to an increased risk of cerebrovascular disease (44). Conversely, the post-HS CSMs also altered correspondingly. Reduced plasma concentrations of L-arginine in patients early after intracerebral hemorrhage were identified as an independent risk factor for poor outcomes (45). Meanwhile, following subarachnoid hemorrhage, nitric oxide metabolites nitrite and nitrate levels are also markedly elevated (46–48). The aforementioned discoveries confirm that CSMs and HS share a reciprocal impact. During our study, we initially identified 73 CSMs that were causally associated with HS; combined with reverse MR, we found similar mannose (carbohydrate), taurochenodeoxycholate (lipid), and tetradecanedioate (lipid), three metabolites that are capable of interfering with ICH. Mannose and taurochenodeoxycholate serve as potential protective factors for ICH, and tetradecanedioate is a potentially hazardous element in ICH. Following the occurrence of ICH, the level of all three rises. Mannose synthesizes glycoproteins and participates in immunomodulation as a widely distributed monosaccharide in body fluids and tissues (49). Hence, it is upregulated after the onset of ICH as a timely energy supplier and immunomodulator. Taurochenodeoxycholate has been linked to the inhibition of cell death (50) and, in turn, may act as a neuroprotective factor that is activated upon the rise of stress following the development of ICH. Regarding tetradecanedioate, the pathway mediating FA β-oxidation, peroxisomal FA β-oxidation or FA α-oxidation may damage vascular components contributing to ICH (51), Whereas, once ICH occurs, it might remain persistently elevated owing to its complex biological functions for example *in vivo* transporter protein assessment of biomarkers (52).

We further adjusted for common confounders of hemorrhagic stroke via multivariate MR analysis, which ultimately identified four metabolites having significant causal effects. To begin with, biliverdin (cofactors and vitamins) was characterized as an ICH protective element. It acts as a by-product of heme degradation, and numerous studies have confirmed that its biological functions are tightly linked to anti-inflammatory, anti-apoptotic, and anti-oxidative stress (53). Moreover, it also inhibits the expression of the pro-inflammatory cytokines interleukin 1β, tumor necrosis factor α, and interleukin 6 to achieve an anti-inflammatory role (54). Studies have shown that the anti-oxidative stress effect of biliverdin (cofactors and vitamins) can be accomplished by scavenging superoxide (55); meanwhile, biliverdin protects vascular tissue from vessel damage by reducing c-Jun NH2 terminal kinase activation and preventing endothelial cell apoptosis (56); additionally, it regulates the Nrf2/A20/eEF1A2 axis to suppress cellular death and thereby attenuates cerebral ischemia–reperfusion injury (57). However, the main mechanisms involved in the pathogenesis of ICH include damage to the cerebral vascular wall, vitellosis, and lipid deposition (4); in the latest study, oxidative stress erythrocyte-associated erythrocyte-brain endothelial interactions that can induce microglia activation *in vivo* also lead to cerebral hemorrhage (58). In summary, biliverdin probably blocks the occurrence and progression of ICH by decreasing vascular inflammation and cellular pyroptosis caused by the release of cellular inflammatory factors, preventing vascular endothelial apoptosis and oxidative stress in erythrocytes. Linoleate (18:2n6) (lipid) is likewise recognized as a protection factor for ICH by us, and it is an n-6 polyunsaturated fatty acid essential for average growth and development. It is found in low concentrations within the brain (<2% of total fatty acids). It constitutes a necessary precursor to benign fatty acids and arachidonic acid in the brain, which has important implications for neurodevelopment and the regulation of pain and inflammatory signaling in peripheral tissues (59). The bio function of linoleate (18:2n6) is more sophisticated. There is a belief that long-term consumption of a low-level linoleate diet might protect the brain from inflammation. It has been found that a dietary structure in which linoleate is completely deprived for an extended period leads to a rapid loss of the beneficial components of ceruloplasmin in the rat brain and, conversely, an accumulation of anti-inflammatory lipids in the rat brain following low intake of linoleate. In contrast, excessive intake promotes neuroinflammation in the rats (60, 61). The genotype is closely related to how linoleate exerts inflammatory and metabolic responses in the human body (62). Briefly, linoleate (18:2n6) may act as an ICH protective factor through an anti-inflammatory mechanism of action, but long-term low-level intake is required to fulfill this conclusion. When ingested in excess over time, it could become a risk factor for ICH by enhancing the inflammatory response. Therefore, more in-depth mechanisms need to be investigated further.

SAH usually occurs in association with aneurysm rupture, and the mechanisms behind it are primarily hemodynamic stress and vascular wall injury with inhibition of its repair (5). Apart from the significant discovery of ICH and circulating serum metabolites, we have also identified circulating serum metabolites relevant to SAH. Our analysis showed that 1-eicosadienoylglycerophosphocholine* (lipid) promoted SAH. 1-eicosadienoylglycerophosphocholine*, alias lysophosphatidylcholine (20:2), a cleavage product of phosphatidylcholine, and in addition, lysophosphatidylcholine (20:2) (LPC) plays a critical role in promoting the progression of atherosclerosis and other cardiovascular diseases by affecting endothelial cells, vascular smooth muscle cells, and arteries (63). LPC is recognized as a significant component of oxidatively damaged low-density lipoprotein (oxLDL), which induces migration of lymphocytes and macrophages, increases the production of pro-inflammatory cytokines, and can aggregate inflammation, trigger oxidative stress, and promote apoptosis. On the other hand, it activates NLRP3 and NLRC4 inflammatory vesicles in microglia and astrocytes. It also synergizes with Procaspase-1 to induce activation of the ROS promoter CYP1B1 and a robust inflammatory response in human arterial endothelial cells (64, 65). Furthermore, lysophosphatidylcholine (20:2), besides inducing oxidative stress in human endothelial cells through NOX5-mediated increases in intracellular calcium (66), also activated voltage-dependent calcium channels to increase calcium influx and enhanced 5-HT-induced contraction of vascular smooth muscle cells in umbilical arteries (67). These micro-mechanisms provide more evidence that LPC can result in cerebral neurovascular inflammatory injury, oxidative stress, and rapid hemodynamic changes, consistent with our results. Hence, elevated levels of 1-eicosadienoylglycerophosphocholine* are likely to be the mechanism behind the development of SAH. 7-Hoca is a naturally existing cholesterol metabolite in human blood, produced by the metabolism of 7α-hydroxy-4-cholestene-3-one and 27-hydroxycholesterol in the brain. It is also an intermediate metabolite in the synthesis of bile acids (BA) (68). A significant rise in the concentration of 7-Hoca after SAH was observed (69); while the conversion of 27-hydroxycholesterol to 7-Hoca is an essential mechanism for the elimination of preoxidized sterols of the brain, 27-hydroxycholesterol has been implicated in the protection of both brain and cognition after injury (70). From this, the increase in 7-Hoca after SAH may indicate accelerated metabolism of 27-hydroxycholesterol to mediate cerebral protection. BA, a downstream product of 7-Hoca, could lower plasma triglycerides by inhibiting hepatic SREBP-1c expression or modulating glucose-induced adipogenesis. Meanwhile, BA’s activation of the farnesoid X receptor (FXR) improved dyslipidemia in mice (71). So, 7-Hoca might be a metabolite acting as a cerebroprotective agent. Therefore, we consider that 7-Hoca exerts a potential protective function in SAH primarily via abolishing 27-hydroxycholesterol and facilitating BA as a downstream product, thereby mediating lipid regulation. Yet more direct mechanisms of action require more thorough exploration.

Although we have done complete and systematic research, some limitations remain. Firstly, the sources we chose for the dataset are all European, with results that are not guaranteed in terms of population generalizability. Secondly, to obtain a sufficient number of SNPs for MR analysis of the exposure data, we slightly liberalized the filtering criteria; this may have made the IVs less effective, although it is common practice among other studies, And the fact that the IVs we got are all strong instrumental variables and the directionality test has no wrong causal direction also strengthens the credibility of our results in some extent. Lastly, we have examined many metabolites of recognized functional structure, yet many unknown circulating serum metabolites could not be researched. Nevertheless, we went through a series of rigorous and comprehensive MR analyses, which led to the identification of a reliable causal relationship, which can provide a high reference value for carrying out more in-depth interaction research between CSMs and HS.

## 5 Conclusion

In conclusion, we comprehensively and systematically evaluated the causality among circulating serum metabolites and hemorrhagic stroke utilizing MR analysis. Four circulating serum metabolites with statistically significant and robust causal effects with hemorrhagic stroke were eventually identified. Of these, biliverdin and linoleate (18:2n6) are capable of decreasing the risk of ICH, 1-eicosadienoylglycerophosphocholine* is a hazard factor for SAH and 7-Hoca is a protective element for SAH, the mediation of inflammation, oxidative stress, apoptosis, lipid homeostasis and hemodynamics are the likely mechanisms for the action of these metabolites. Additionally, another ten notable metabolic pathways have been characterized. These results suggest that these metabolites might be considered biomarkers for HS prevention and monitoring and also provide some references and assistance for future research on the selection of circulating metabolites and the exploration of the mechanism of preventive and curative targets.

## Data availability statement

The original contributions presented in the study are included in the article/Supplementary material, further inquiries can be directed to the corresponding authors.

## Author contributions

YW: Conceptualization, Investigation, Visualization, Writing – original draft, Writing – review & editing. YS: Data curation, Software, Visualization, Writing – original draft, Writing – review & editing. QL: Resources, Software, Writing – review & editing. HX: Data curation, Investigation, Resources, Writing – review & editing. AG: Data curation, Investigation, Methodology, Writing – original draft. KL: Conceptualization, Investigation, Software, Writing – review & editing. YR: Data curation, Methodology, Resources, Writing – review & editing. SG: Methodology, Resources, Writing – review & editing. HL: Funding acquisition, Supervision, Writing – review & editing. XZ: Funding acquisition, Supervision, Writing – review & editing.

## Footnotes

^1^ http://metabolomics.helmholtz-muenchen.de/gwas/

^2^ https://www.metaboanalyst.ca/
